# Predictability of crowding resolution in clear aligner treatment

**DOI:** 10.1186/s40510-022-00438-z

**Published:** 2022-11-28

**Authors:** Adriana Fiori, Giuseppe Minervini, Ludovica Nucci, Fabrizia d’Apuzzo, Letizia Perillo, Vincenzo Grassia

**Affiliations:** Multidisciplinary Department of Medical-Surgical and Dental Specialties, University of Campania Luigi Vanvitelli, Via Luigi De Crecchio 6, 80138 Naples, Italy

## Abstract

**Background:**

To assess the predictability of crowding resolution and the efficacy of different strategies to gain space during clear aligners treatment.

**Methods:**

A total of 10 clinicians were randomly recruited using the Doctor Locator by Align Technology (California). For each clinician, four consecutive patients treated with aligners and manual stripping were selected for a total of 40 subjects. Thus, 80 arches were collected and uploaded on the Orthoanalyzer software for arch measurements. The data were gained on the starting arch form (T0), on the virtual arch developed with digital planning (vT1), and on the arch form achieved at the end of the aligner sequences (T1). The following parameters were scored: Little's Irregularity Index, transversal arch diameters, (intercuspid, interpremolar, and intermolar width), incisor position/arch length, and enamel interproximal reduction (IPR).

**Results:**

For all the measurements, statistically significant differences were found at different stages. The predictability of crowding resolution was very high, ranging from 87% in the upper arch and 81% in the lower one. Among the different strategies to gain space, variations in sagittal incisor position were predictable, with a value of 70% both in the upper and lower arch. Conversely, changes in arch diameters were less reliable varying between 49 and 67% in the lower arch and 59–83% in the upper one. Moreover, IPR was the least accurate procedure, wavering at 49% in the upper arch and 42% in the lower arch.

**Conclusions:**

The predictability of crowding resolution during treatment with aligners was high. However, the virtual arch forms obtained at the end of digital planning (vT1) did not correspond with the arch forms at the end of the aligner sequences (T1). The IPR was the least predictable strategy to gain space, being, perhaps, an operator-dependent procedure.

## Background

From the initial spread of clear aligners in the orthodontic field, their application was intended for mild crowding. With the development of the material and the computer design of the tooth movement, the indication of clear aligners has been broadened to moderate or severe malocclusion, including extraction or non-extraction cases [1].

However, in the extraction cases approached with clear aligners, molar anchorage control and central incisor retraction were not achieved as predicted, getting body movements with this technique is still difficult, although the use of elastic chains and other auxiliaries could improve them [2, 3].

Clear aligners have improved the patients’ esthetics, comfort, and hygiene. On the other hand, they have some limits in controlling some tooth movements [4].

Since these new devices were introduced as a therapeutical option for orthodontic treatment, orthodontists have been investigating the effectiveness of the treatment and the accuracy of tooth movements, comparing the predicted with the achieved result through digital models. Aligners have the advantage of perspective three-dimensional digital planning and the disadvantage of the limited predictability of some dental movements [3–9].

For treatments with aligners to be effective, there must be a correspondence between the planned and actual results. All the studies on this topic agree that in most cases, there are some discrepancies between the digital setup and the clinical result [5, 9].

According to Buschang, virtual models do not accurately reflect the patients’ final occlusion immediately at the end of active treatment: they overestimated alignment, rotations with occlusal contacts, and occlusal relations [10].

As reported in a recent Systematic Review [11], in almost all cases, additional refinements are needed to achieve the prescribed setup results. In other words, the number of patients requiring an unplanned correction or even resorting to fixed orthodontics to get the results indicated in the virtual model is closer to 70–80% [5].

The crowding resolution with clear aligner is possible by proclining the tooth, expanding the arch, performing IPR, or, in the most serious cases, extracting, thus using the same approaches used for the conventional orthodontic treatment [10, 11], however, it is less predictable with aligners to obtain pure translation movements in extraction cases [2].

In 2012, Krieger et al. [12] assessed arch length, intercuspid distance, overbite, overjet and midlines by comparing initial and final casts, which were measured with a clinical caliper. They provided a general conclusion that clear aligner treatment effectively resolved anterior crowding by incisor proclination, but overbite correction was difficult to achieve [13].

However, the expansion efficiency decreased from the canine to the first molar [14–16], while the amount of enamel removed in vivo did not correspond with the amount of IPR planned [17, 18].

Knowing the accuracy of the software in predicting tooth movements could help clinicians to overcorrect if necessary or staging the movement in smaller increments, thereby reducing refinements, mid-course corrections, and treatment time. So orthodontists must be fully aware of the features and weaknesses of this device in order to choose the correct indications and obtain good treatment results [19–21].

The current study aims to assess the predictability of crowding resolution and the efficacy of the different strategies to gain space during clear aligner treatment and their correlation to provide a suitable protocol to achieve predictable results.

## Materials and methods

This retrospective observational study was approved by the Institutional Review Board of the University of Campania *Luigi Vanvitelli*. (No. 308 20/512019).

### Subject recruitment

The sample size was estimated based on preliminary data [17]. A minimum sample of 39 subjects was needed to achieve 80% power, with an alpha of 5% to detect a 0.5 mm difference (SD 0.5 mm).

The sample was obtained from 10 orthodontists selected using the Doctor Locator (DL) with the method and inclusion criteria described in the previous paper [17]:

1. at least 8 years of experience in CAT;
2. execution of manual IPR;
3. at least 100 patients were treated with CA last year.

Ten Italian ZIP codes have been randomly drawn and entered into the DL application. The first ten doctors that agreed to participate were included in the provider's list in that area.

For each doctor, the last four consecutive patients treated with clear aligner were selected according to the following inclusion criteria:

1. adult patients with full permanent dentition;
2. non-extraction orthodontic treatment in both arches;
3. use of composite attachments;
4. manual IPR digitally planned (between 0.1 mm and 0.5 mm per tooth);
5. virtual digital planning with Clincheck (Invisalign; Align Technology, Santa Clara, Calif);
6. digital models at the beginning of treatment and after the first aligner sequence;
7. good compliance with aligner;
8. treatment started in 2014 or later (after the introduction of the SmartTrack material).

Exclusion criteria were as follows:

1. interruption of aligner sequence;
2. poor compliance;
3. multiple and/or advanced caries;
4. supernumerary teeth;
5. cleft lip and/or palate.

The orthodontist placed the attachments with no restrictions, according to his preferences. Aligners were changed on average every 10 days.

For each patient, we evaluate the predictability of the three different strategies to gain space:

1. transversal arch expansion;
2. arch length;
3. interproximal enamel reduction.

### 3D casts and measurement protocol

Records were collected from the Invisalign Doctor Web Site. Digital models were exported as STL (Standard Triangulation Language) files at the beginning (T0), at the end of the virtual digital planning (vT1), and at the end of the corresponding aligner sequence (T1) and uploaded into OrthoAnalyzer Program (version 1.7.1.4; 3Shape, Copenhagen, Denmark).

**Fig. 1:**
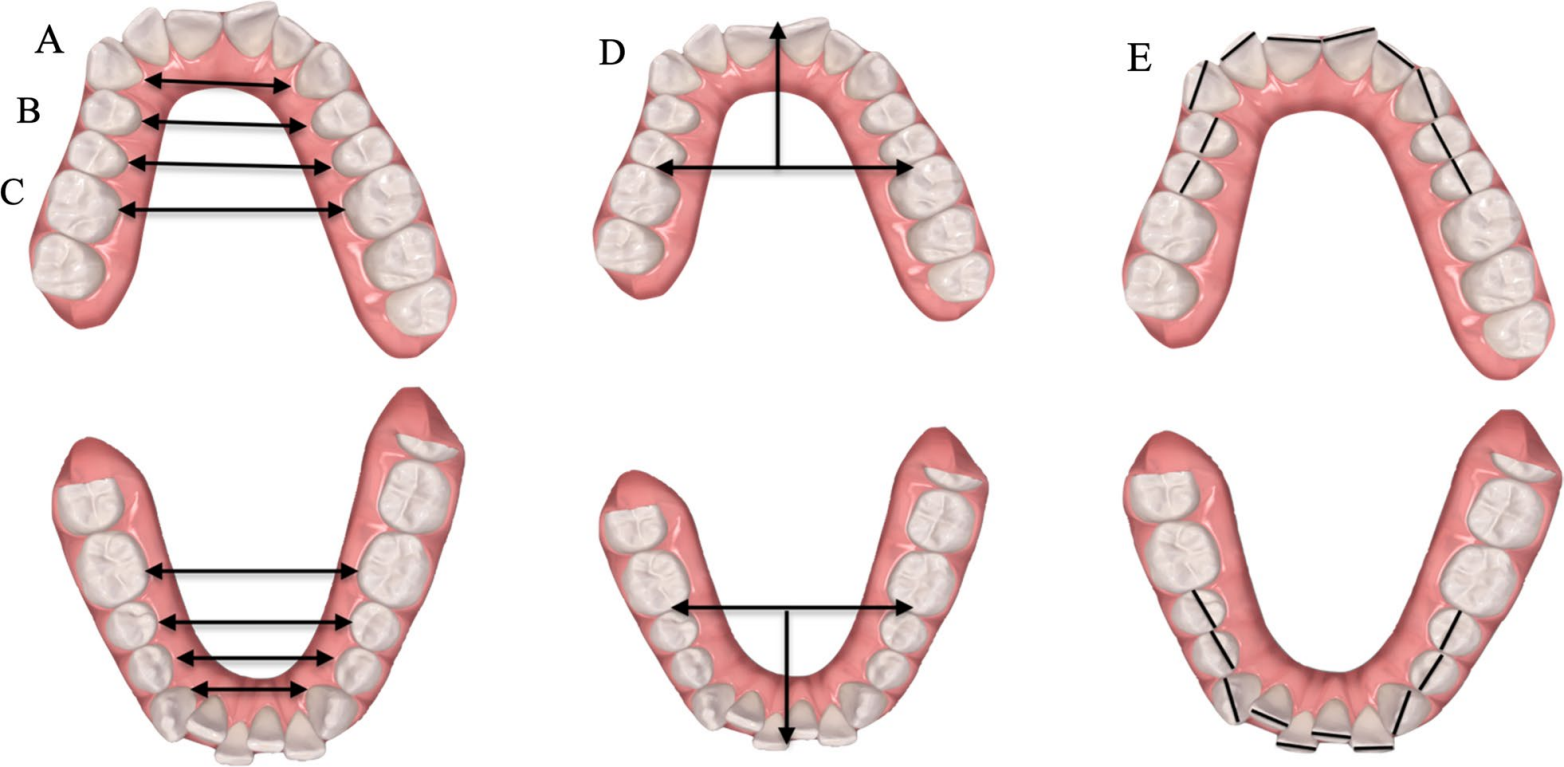
Measurements: upper and lower arch widths, arch length, and interproximal enamel reduction. A–C Intercuspid width: linear distance between inner lingual points; interpremolar width: linear distance between inner lingual points; intermolar width: linear distance between inner lingual points; D arch length: the perpendicular to a line connecting the contact point passing through the buccal point; E Interproximal enamel reduction: the difference between mesiodistal diameter at T0 and T1

An operator (AF) defines for each tooth, from the first molar to molar, all landmarks illustrated in Table 1 to be able to carry out all the measurements (Fig. 1).

**Table 1:** Landmarks

Landmarks	Construction and Identification methods
Tooth long axis	Through the plane that divides the tooth into two equal parts
Tooth mesiodistal diameter	Through the line connecting the most mesial and distal points of the crown
Lingual/palatal inner point	The most apical point proximally to the gingival marginal of each tooth on the lingual/palatal side
Buccal point	The most facial point on the most prominent central incisor
Contact point	The point between the second premolar and the first molar

The arch measurements were performed, similarly to those proposed by Raucci [22], to evaluate the changes between the three-time steps (T0; vT1; T1) (Fig. 1).

Then for each arch to evaluate the real IPR, the mesiodistal teeth dimensions were measured from second-to-second premolar before and after IPR (Fig. 1E). The full-arch amount of IPR performed was obtained through the difference between the length of mesiodistal tooth diameters before and after treatment [17].

After it was compared to the planned IPR, shown in the virtual digital planning.

Dental crowding was quantified by Little’s Irregularity Index [23].

The following formula was used to quantify the predictability of each measurement:

predictability: [real post-treatment (T1)—initial pre-treatment (T0)]/[ideal post-treatment (vT1)—initial pre-treatment (T0)].

Thus, an index of the predictability of each strategy was obtained: the closer the value to 1, the more precise strategy was performed by the aligner (100% of the prescription).

To evaluate the correlation between each space gain strategy and crowding resolution, the treatment outcomes were assessed through the measurement changes between the start (T1) and end of treatment (T0) (Delta: Δ).

### Statistical analysis

The statistical analysis was performed with Jamovi 2.3.2 statistical software (The jamovi project, Sidney, Australia). Descriptive statistics were calculated for each measurement. The data distribution was normal, so a paired t test to determine if there was a statistically significant change between vT1 and T1 (*p* < 0.05) was used.

To assess reliability, 1 month after the initial measurements, the parameters were remeasured by the same examiner. Intraclass correlation coefficients showed moderate interobserver reliability, with Cronbach's alpha of 0.723 for linear measurements.

In the presence of normally distributed data, a paired t test was used to compare the T1-T0 changes and the vT1-T0 changes. The level of significance was set at 5%.

A Pearson's product-moment correlation was run to assess the correlation between the difference of Irregularity Index (before and after treatment and the outcomes of strategies to gain space.

## Results

A total of 40 adult Caucasian subjects (18 men, 22 women, ﻿of mean age 34 ± 7) for a complete of 80 dental arches were achieved.

All the patients completed treatment in keeping with the research protocol. Patient compliance forms were collected at the tip of treatment; all patients reported wearing their aligners for 21 to 23 h per day. For all measurements, statistically significant differences were found at the various stages as reported in Table 2.

**Table 2:** Comparison between T0, T1v, and T1 in upper arch

Variables	T0		v T1		T1		Shapiro–Wilk	Predictability (%)	*p*
	Mean	SD	Mean	SD	Mean	SD			
Irregularity Index	7.26	3.30	1.83	0.65	2.81	1.72	0.40	87.0	0.04*
3-3 width	23.53	2.55	24.83	2.01	24.20	1.95	0.90	59.0	0.03*
4-4 width	26.08	2.50	28.10	2.40	27.20	2.26	0.96	60.0	0.02*
5-5 width	30.74	2.62	32.67	2.67	31.93	2.60	0.77	68.0	0.01*
6-6 width	33.24	3.21	34.74	2.93	34.40	2.94	0.86	83.0	0.00*
Arch length	28.40	2.29	27.27	2.17	27.67	2.31	0.93	68.0	0.04*
IPR	–	–	1.09	1.13	0.55	0.64	0.85	49.0	0.02*

*p < 0.05

Preliminary analyses showed the link to be linear with both variables normally distributed, as assessed by Shapiro–Wilk's test (*p* > 0.05),

The predictability of crowding resolution measured with the decrease in the Irregularity Index was very high, starting from 87% (*p* < 0.04) within the upper arch and 81% (*p* < 0.03) within the lower one.

Conversely, changes in arch diameters were less reliable varying between 59 and 83% within the upper arch and 49–67% within the lower one (Tables 2, 3).

In the upper arch, the foremost accurate prediction was for the first molar with the predictability of 83% (*p* < 0.00), while the smallest amount was for the canine of 59% (*p* < 0.03) (Table 2).

Moreover, within the lower arch, the foremost accurate prediction was for the second premolar with the predictability of 68% (*p* < 0.09), while the smallest amount was for the canine at 49% (*p* < 0.03) (Table 3).

**Table 3:** Comparison between T0, T1v, and T1 in lower arch

Variables	T0		v T1		T1		Shapiro–Wilk	Predictability (%)	*p*
	Mean	SD	Mean	SD	Mean	SD			
Irregularity index	8.13	4.10	1.40	0.30	2.66	1.57	0.89	81.0	0.03*
3-3 width	18.48	1.82	19.92	1.79	19.15	1.42	0.94	49.0	0.03*
4-4 width	25.21	2.35	26.38	1.96	26.03	1.83	0.91	67.0	0.02*
5-5 width	29.60	2.74	31.10	2.42	31.10	2.42	0.93	68.0	0.09*
6-6 width	32.51	3.11	33.97	2.93	33.37	2.96	0.87	67.0	0.02*
Arch length	24.35	3.10	23.99	3.06	24.00	2.99	0.83	69.0	0.02*
IPR	–	–	1.43	1.10	0.82	0.84	0.89	42.02	0.03*

*p < 0.05

Variations in sagittal incisor position were predictable, with a worth of 68% within the upper arch (*p* < 0.04), and 69% (*p* < 0.02) within the lower one (Table 4).

**Table 4:** Summary of the variables' predictabilities

	Irregularity index (%)	Arch widths (%)	Arch length (%)	IPR (%)
Upper	87	59–83	68	49
Lower	81	49–67	69	42

Lastly, IPR was the smallest amount accurate procedure, wavering from 49% within the upper arch and 42% within the lower arch.

**Fig. 2:**
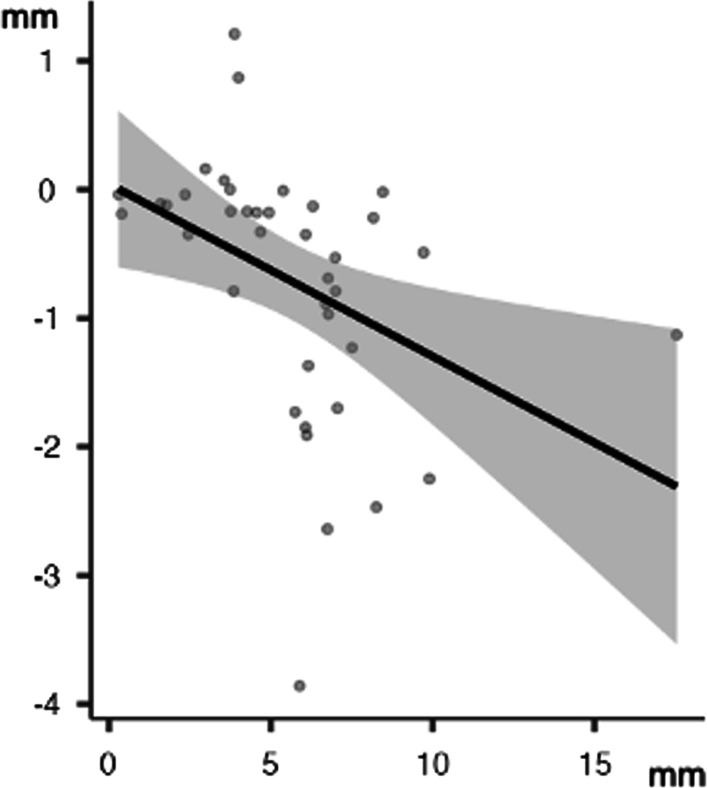
Lower arch: linear correlation between Δ irregularity index and Δ 3-3

**Fig. 3:**
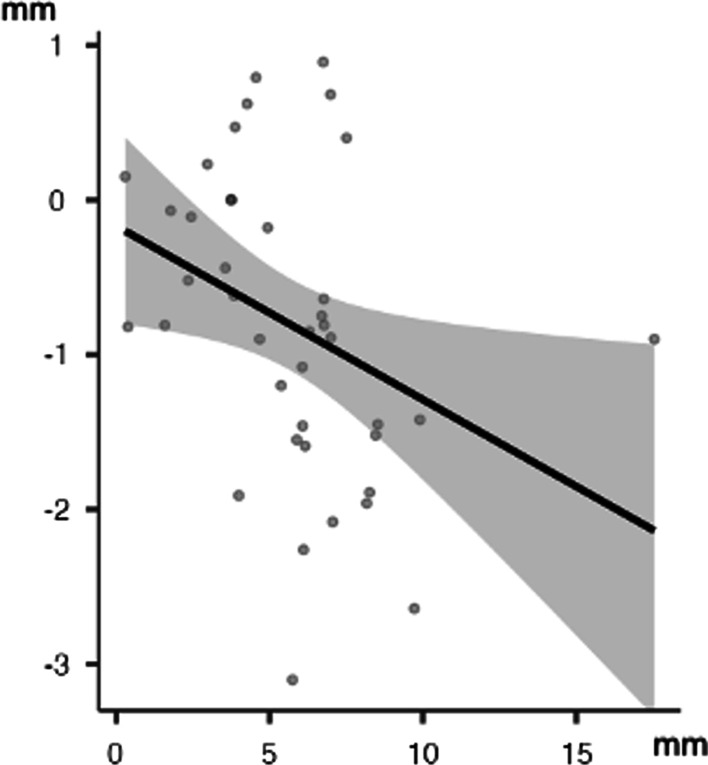
Lower arch: linear correlation between Δ irregularity index and Δ 4-4

In the lower arch, a Pearson's product-moment correlation was run to assess the relationship between the different strategies to gain space and Irregularity Index. There was a statistically significant, high inverse correlation between arch width (Δ 3-3) and Δ Irregularity Index, (*r* = −0.41; *p* < 0.05) (Fig. 2). Moreover, there was a statistically significant, moderate inverse correlation between arch width (Δ 4-4) and Δ Irregularity Index, (*r* = −0.35; *p* < 0.05) (Fig. 3).

There was no statistically significant correlation between the other strategies and the Irregularity Index (Table 5).

**Table 5:** Correlation between space gain strategy and crowding in upper arch

Δ Irregularity index	Δ 3-3	Δ 4-4	Δ 5-5	Δ 6-6	Δ Arch length	Δ IPR
Pearson's *r*	− 0.41	− 0.35	− 0.21	− 0.27	0.09	0.31
*p* value	0.01	0.03	0.19	0.12	0.58	0.06

**Fig. 4:**
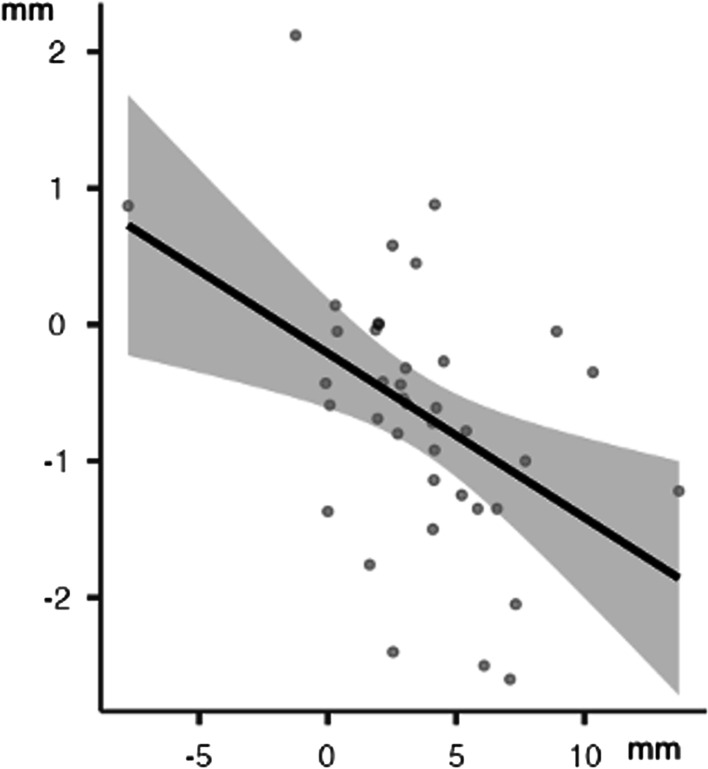
Upper arch: linear correlation between Δ irregularity index and Δ 4-4

Moreover, in the upper arch, there was a statistically significant, high inverse correlation between arch width (Δ 3-3) and Δ Irregularity Index, (*r* = −0.45; *p* < 0.05) (Fig. 4).

There was no statistically significant correlation between the other strategies and the Irregularity Index (Table 6).

**Table 6:** Correlation between space gain strategy and crowding in the upper arch

Δ Irregularity index	Δ 3-3	Δ 4-4	Δ 5-5	Δ 6-6	Δ Arch length	Δ IPR
Pearson's *r*	− 0.45	− 0.27	0.04	− 0.04	0.08	0.03
*p* value	0.01	0.09	0.80	0.80	0.63	0.88

## Discussion

The aim of this investigation was to assess the predictability of the crowding resolution, the efficacy of the different strategies to gain space, and their correlation to provide a suitable protocol to achieve predictable results.

The sample of this study presented an Irregularity Index mean of 7.26 mm in the upper arch and 8.13 mm in the lower one. These data indicated that the patients recruited had severe crowding in both arches, suggesting orthodontists' confidence in treating this issue with clear aligners.

At the end of digital planning (vT1), a residual crowding of 1.83 mm in the upper arch and 1.40 in the lower arch was still present. Therefore, it could be deduced that planning errors are often present and could affect the final outcomes.

At the end of the first set of aligners (T1), the crowding was statistically significantly reduced, but an Irregularity Index of 2.81 mm in the upper arch and 2.66 mm in the lower arch was still found. These data show a high value of predictability (87% in the upper arch and 81% in the lower one), confirming that the clear aligner treatment is efficacy to reduce crowding [24]. Unfortunately, single planning, even if very effective, is not enough to solve the crowding entirely.

Several studies compare, in clear aligner patients, the treatment outcomes that needed refinement, with their digital planning to evaluate the effectiveness of tooth movement. The results of these researches were similar to ours [12, 15, 23].

Therefore, additional aligners should always be considered to solve the crowding still present.

The predictability of crowding resolution with clear aligners is a multifactorial issue. Some are related to aligners, such as the protocol used, the features and the thickness of the aligner material, the planning software, and the different strategies to gain space planned.

Others depend on the operator, such as the experience of the clinician in case selection [19], the accuracy plan of the strategies to gain space, and the accuracy of IPR procedures [17].

As emerged in this paper, the single strategies to gain space reached different predictability, although they together, concur to solve crowding. Our results showed large variability of these data.

For example, transverse changes in the upper arch ranged between 59 and 83% depending on the form of the diameters considered.

This indicates that the aligners do not allow the planned expansion entirely as already reported in the literature [20, 21]. The least accurate transversal diameter change, as also reported in previous studies [15, 16, 20, 25], was for canine and the first premolar with the predictability of 59% and 60%, while the most accurate was for the first molar with the predictability of 83%. This could be related to the different extensions of the lingual surface and anatomy of each tooth related to the retention and fit of the clear aligner. Indeed, the slender and conical canine shape could not help to control the planned expansion movement.

In the mandibular arch, the predictability is even lesser, with the lowest value always found for the canine diameter of 49%. Mandibular canines have the longest root, a shape of crown with few undercuts, and a small lingual surface due to their usual more vestibular position; this reduces the retention of the aligner and the ability to push the tooth buccally.

The data regarding the sagittal incisor position (arch length) indicate that, on average, at the end of virtual digital planning (vT1), control of the arch length is planned [12, 26].

Instead, at T1, the slightly lingual movement of the incisors, through coronal tipping, was predictable with a value of about 70% both in the upper and lower arch.

Also, Kravitz reported [27], that the aligners are more accurate in retracting the incisor than in expanding them labially.

Obviously, arch length decrease or remaining stable is associated with the posterior expansion of the dental arch and it might be due to the maxillary arch form [28].

These results highlight that with the clear aligners, it is possible to plan crowding resolution without buccal tipping, thus avoiding side effects of the lower incisors proclination, which are often present with fixed appliances.

The clinicians should take in mind these outcomes to plan crowding resolution with less canine expansion and incisor proclination but primarily with IPR.

Regarding IPR, after aligner sequences, in the upper arch, less than half, 0.55 mm, was performed, while in the lower arch the result was better, 0.82 mm. These data attest to the accuracy of IPR at 49% in the upper arch and 42% in the lower one, values that confirm the results of the previous study [17].

These data indicate that in these patients, less than half of the planned IPR at the start of treatment with software was performed.

The predictability of IPR is a multifactorial issue. The amount of enamel removed depends, in fact, on several factors: some related to characteristics of the tooth such as the hardness of the enamel, the anatomy of the crown, or the position of the tooth itself. Others related, instead, to the operator, such as his experience, the technique used for the IPR, and the pressure exerted during the procedure [17].

Therefore, IPR is the least accurate strategy to gain space, and it requires greater accuracy. This could improve the predictability of crowding resolution, reaching the optimal values planned.

Moreover, there was not a high correlation between the irregularity index and the strategies to gain space; however, all the strategies concur to create useful space to solve the crowding.

In both arches, a high inverse statistically significant correlation was found between intercuspid diameters (Δ 3-3) and Δ Irregularity Index. This result indicates that crowding decreases as 3-3 diameter increases.

Incisor proclination (Δ arch length) was not correlated to Δ Irregularity Index, obviously because in digital planning there was no proclination to gain space.

Moreover, the correlation between Δ IPR and Δ Irregularity Index was positive, although not statistically significant. Indeed, with the reduction in enamel increasing, we registered a resolution of crowding improvement, although IPR has rather low efficacy rates. The result of this research showed that digital planning was not a predictor of final tooth position. Therefore, the virtual tooth position may not be the achieved final tooth position. So, it is important to know the limits of digital planning to overcorrect tooth movements when it is necessary to decrease the need for refinement [12]. Knowing the strengths and the weaknesses of clear aligners will help the clinician in selecting the best orthodontic appliance to treat a specific malocclusion.

Therefore, orthodontists play an important role in modifying the virtual digital plan with their clinical experience, programming the movements at the proper steps, and adding the features to improve the predictability of the tooth movement with the aligners [29].

To prevent the risk of selection bias, due to the retrospective nature of the study, patients were consecutively selected for each orthodontist. This study included only adult patients to avoid bias due to normal transverse growth of the jaws and because they currently represent most of the patients who request orthodontic treatment with clear aligners. Moreover, these patients generally show better compliance compared to adolescents, thus reducing a possible source of bias [9, 17].

However, the retrospective studies do not allow for estimating precisely patient cooperation. Thus, to verify the collaboration of the patients, their charts were reviewed at the end of the treatment to check if the aligners had been changed at regular intervals, but this may have been influenced by patient statements.

Future studies should add other measurements such as lateral cephalometric or volumetric 3-dimensional cone-beam to assess predictability of the different strategies to gain space. Such studies will allow for the evaluation of posterior tooth movement and address questions regarding root movement with clear aligners. Obviously, the possibility of developing prospective randomized clinical trials would allow emerging greater certainty regarding the predictability of treatment with clear aligners.

## Conclusions

This study showed that the predictability of crowding resolution with clear aligners is 87% in the upper arch and 81% in the lower one.

Transversal arch expansion's predictability achieves a value between 59 and 83% in the upper arch and 49–67% in the lower one, decreasing from molars to canines in both arches.

Sagittal arch length is less predictable, and it presents the same value in both arches.

IPR is the least accurate strategy to gain space.

A correlation between the three gain space strategies and crowding was found only with the increase in arch transversal diameters. Specifically, the changes in 3-3 diameters were correlated with crowding resolution both in the upper and lower arch, whereas 4-4 only in the upper one.

## Data Availability

The datasets used and/or analyzed during the current study are available from the corresponding author on reasonable request.

## References

[1] Ke Y, Zhu Y, Zhu M (2019). A comparison of treatment effectiveness between clear aligner and fixed appliance therapies. BMC Oral Health.

[2] Dai F, Xu T, Shu G (2021). Comparison of achieved and predicted crown movement in adults after 4 first premolar extraction treatment with Invisalign. Am J Orthod Dentofac Orthop.

[3] Lombardo L, Palone M, Maino G, Paoletto E, Carlucci A, Siciliani G (2021). Class II subdivision with skeletal transverse maxillary deficit treated by single-sitting bone-borne appliance: a case report. Angle Orthod.

[4] Azaripour A, Weusmann J, Mahmoodi B, Peppas D, Gerhold-Ay A, Van Noorden CJF (2015). Braces versus Invisalign®: Gingival parameters and patients’ satisfaction during treatment: a cross-sectional study. BMC Oral Health.

[5] Lombardo L, Arreghini A, Ramina F, Huanca Ghislanzoni LT, Siciliani G (2017). Predictability of orthodontic movement with orthodontic aligners: a retrospective study. Prog Orthod.

[6] Rossini G, Parrini S, Castroflorio T, Deregibus A, Debernardi CL (2015). Efficacy of clear aligners in controlling orthodontic tooth movement: a systematic review. Angle Orthod.

[7] Papadimitriou A, Mousoulea S, Gkantidis N, Kloukos D (2018). Clinical effectiveness of Invisalign® orthodontic treatment: a systematic review. Prog Orthod.

[8] Galan-Lopez L, Barcia-Gonzalez J, Plasencia E (2019). A systematic review of the accuracy and efficiency of dental movements with invisalign®. Korean J Orthod.

[9] Tepedino M, Paoloni V, Cozza P, Chimenti C (2018). Movement of anterior teeth using clear aligners: a three-dimensional, retrospective evaluation. Prog Orthod.

[10] Buschang PH, Ross M, Shaw SG, Crosby D, Campbell PM (2015). Predicted and actual end-of-treatment occlusion produced with aligner therapy. Angle Orthod.

[11] Robertson L, Kaur H, Fagundes NCF, Romanyk D, Major P, Flores MC (2020). Effectiveness of clear aligner therapy for orthodontic treatment: a systematic review. Orthod Craniofac Res.

[12] Krieger E, Seiferth J, Marinello I, Jung BA, Wriedt S, Jacobs C (2012). Invisalign®-Behandlungen im Frontzahnbereich: Wurden die vorhergesagten Zahnbewegungen erreicht?. J Orofac Orthop.

[13] Haouili N, Kravitz ND, Vaid NR, Ferguson DJ, Makki L (2020). Has Invisalign improved? A prospective follow-up study on the efficacy of tooth movement with Invisalign. Am J Orthod Dentofac Orthop.

[14] Zhou N, Guo J (2020). Efficiency of upper arch expansion with the Invisalign system. Angle Orthod.

[15] Houle JP, Piedade L, Todescan R, Pinheiro FHSL (2017). The predictability of transverse changes with Invisalign. Angle Orthod.

[16] Lione R, Paoloni V, Bartolommei L, Gazzani F, Meuli S, Pavoni C (2021). Maxillary arch development with Invisalign system. Angle Orthod.

[17] De Felice ME, Nucci L, Fiori A, Flores-Mir C, Perillo L, Grassia V (2020). Accuracy of interproximal enamel reduction during clear aligner treatment. Prog Orthod.

[18] Kalemaj Z, Levrini L (2021). Quantitative evaluation of implemented interproximal enamel reduction during aligner therapy: a prospective observational study. Angle Orthod.

[19] d’Apuzzo F, Perillo L, Carrico CK, Castroflorio T, Grassia V, Lindauer SJ (2019). Clear aligner treatment: different perspectives between orthodontists and general dentists. Prog Orthod.

[20] Solano-Mendoza B, Sonnemberg B, Solano-Reina E, Iglesias-Linares A (2017). How effective is the Invisalign® system in expansion movement with Ex30′ aligners?. Clin Oral Investig.

[21] Morales-Burruezo I, Gandía-Franco JL, Cobo J, Vela-Hernández A, Bellot-Arcís C (2020). Arch expansion with the invisalign system: efficacy and predictability. PLoS ONE.

[22] Raucci G, Elyasi M, Pachêco-Pereira C, Grassia V, d’Apuzzo F, Flores-Mir C, et al. Predictors of long-term stability of maxillary dental arch dimensions in patients treated with a transpalatal arch followed by fixed appliances. Prog Orthod. 2015. 10.1186/s40510-015-0094-9. 10.1186/s40510-015-0094-9 PMC4516145 26215180

[23] Little RM (1975). The irregularity index: a quantitative score of mandibular anterior alignment. Am J Orthod.

[24] Kassam SK, Stoops FR (2020). Are clear aligners as effective as conventional fixed appliances?. Evid Based Dent.

[25] Charalampakis O, Iliadi A, Ueno H, Oliver DR, Kim KB (2018). Accuracy of clear aligners: a retrospective study of patients who needed refinement. Am J Orthod Dentofac Orthop.

[26] Duncan LO, Piedade L, Lekic M, Cunha RS, Wiltshire WA (2016). Changes in mandibular incisor position and arch form resulting from Invisalign correction of the crowded dentition treated nonextraction. Angle Orthod.

[27] Kravitz ND, Kusnoto B, BeGole E, Obrez A, Agran B (2009). How well does Invisalign work? A prospective clinical study evaluating the efficacy of tooth movement with Invisalign. Am J Orthod Dentofac Orthop.

[28] Taner T, Ciǧer S, El H, Germeç D, Es A (2004). Evaluation of dental arch width and form changes after orthodontic treatment and retention with a new computerized method. Am J Orthod Dentofac Orthop.

[29] Best AD, Shroff B, Carrico CK, Lindauer SJ (2017). Treatment management between orthodontists and general practitioners performing clear aligner therapy. Angle Orthod.

